# Impact of Bilateral and Single Internal Thoracic Artery Bypass Grafting on Postoperative Reverse Remodeling in Patients with End-Stage Ischemic Cardiomyopathy

**DOI:** 10.5761/atcs.oa.25-00189

**Published:** 2026-01-15

**Authors:** Yusuke Misumi, Daisuke Yoshioka, Takuji Kawamura, Ai Kawamura, Shin Yajima, Shunsuke Saito, Takashi Yamauchi, Kazuo Shimamura, Shigeru Miyagawa, Osaka Cardiovascular Surgery Research (OSCAR) study group

**Affiliations:** Department of Cardiovascular Surgery, Osaka University Graduate School of Medicine, Suita, Osaka, Japan

**Keywords:** ischemic cardiomyopathy, coronary artery bypass grafting, bilateral internal thoracic artery

## Abstract

**Purpose:**

The internal thoracic artery (ITA) has shown increased production of nitric oxide, which has beneficial effects on ventricular remodeling, among conduits of coronary artery bypass grafting (CABG). However, little is known about the impact of bilateral ITA strategy on postoperative left ventricle (LV) reverse remodeling as compared with single ITA, especially in patients with severely impaired LV function.

**Methods:**

We retrospectively reviewed 126 propensity-matched patients with advanced ischemic cardiomyopathy (ICM) (left ventricular ejection fraction <40%) who underwent isolated multiple CABG utilizing bilateral (BITA group; n = 65) or single (SITA group; n = 61) ITA. The primary endpoint was postoperative reduction in the indexed left ventricular end-systolic volume index (LVESVI). Baseline covariates were adjusted with propensity score-matching.

**Results:**

At baseline, there were no intergroup differences in LVESVI (78 vs. 78 ml/m^2^, P = 0.93) and EuroSCORE II score (3.0% vs. 2.8%, P = 0.70). At 6 months post-surgery, the BITA group reduced LVESVI to a greater degree than the SITA group (−33% vs. −17%, P <0.01), resulting in significantly smaller postoperative LVESVI (49 vs. 63 ml/m^2^, P = 0.03). Multivariable analysis showed that CABG with BITA (P = 0.011) was associated with postoperative LV reverse remodeling.

**Conclusion:**

In patients with ICM undergoing CABG, the in situ BITA strategy was associated with greater reductions in postoperative LV volume.

## Abbreviations

CABG
: coronary artery bypass grafting
ICM
: ischemic cardiomyopathy
ITA
: internal thoracic artery
LV
: left ventricle
LVEF
: left ventricular ejection fraction
LVESVI
: left ventricular end-systolic volume index

## Introduction

Left ventricular (LV) end-systolic volume is a major predictor of survival after myocardial infarction.^1–3)^ Surgical revascularization in patients with ischemic cardiomyopathy (ICM) is a well-established standard therapy with evidence of improved LV function, heart failure symptoms, and prognosis.^4–6)^

Among conduits of coronary artery bypass grafting (CABG), the internal thoracic artery (ITA) has shown superior long-term patency associated with its reduced tendency for spasm and the development of atherosclerosis.^7)^ These features may be explained by the ITA’s peculiar morphologic features, especially in the intima and the media, and its increased production of anti-inflammatory and vasoactive molecules such as nitric oxide.^8–12)^ Interestingly, as well as the protection of conduit from atherosclerotic change, nitric oxide has also shown beneficial effects on ventricular reverse remodeling and cardiac functional recovery.^13–16)^ However, little is known regarding the impact of conduit selection on postoperative cardiac function, especially in patients with severely impaired LV function undergoing CABG.

We hypothesized that in patients with ICM, CABG with bilateral ITA results in greater postoperative LV volume reduction than does CABG with single ITA. To test our hypothesis, we reviewed the impact of bilateral ITA strategy on postoperative LV reverse remodeling and survival in patients with ICM. In this study, “LV remodeling” refers to the dilated state before surgery, while “reverse remodeling” refers to the reduction in LV volume after surgery.

## Materials and Methods

### Patients

This study included 126 patients with ICM (LV ejection fraction [LVEF] ≤40%) who underwent isolated multiple CABG with either bilateral (n = 65) or single (n = 61) ITA grafts between 1995 and 2021 and who had complete transthoracic echocardiogram at 6 months after surgery. All the patients had LV systolic dysfunction and dilation caused by ischemia. Those who underwent concomitant valve surgery or arrhythmia surgery, left ventricular assist device implantation, redo or emergent surgery were excluded from this study. As an impaired endothelial function has been shown for the free ITA graft,^17)^ those who utilized second ITA graft as a free graft were excluded to evaluate the effect of in situ ITA-derived nitric oxide on LV reverse remodeling. Also excluded were those who underwent CABG with single ITA combined with other arterial grafts, which potentially provide conduit-derived nitric oxide.^8)^ Patients’ data were retrieved from the Osaka Cardiovascular Research Group (OSCAR) database, which was approved by the institutional ethics committee of Osaka University Hospital, and written informed consent from each patient was waived for this retrospective study (Reference no. 08218-6; date of approval, January 13, 2015).

### Surgical procedures

All the included patients underwent multiple CABG with single or bilateral ITA grafts. All the ITA grafts were utilized in an in situ fashion. Additional conduit selection, such as the other ITA, radial artery, or saphenous vein, was selected at the respective surgeons’ discretion. The decision between off- and on-pump beating techniques was determined according to both the patients’ clinical profile, including cardiac function and aortic lesions, and the surgeons’ experience.

### Assessment of LV function and degree of mitral regurgitation (MR)

Two-dimensional and Doppler echocardiography procedures were performed prior to surgery (baseline) and 6 months after surgery to assess LV function and MR severity. The severity of MR was graded as 0 (absent), 1+ (trivial), 2+ (mild), 3+ (moderate), or 4+ (severe) based on color Doppler extent and spatial distribution of the regurgitant jet relative to the left atrial area.

### Follow-up and assessment of adverse events

After surgery, the patients were kept on standard heart failure medications, including angiotensin-converting enzyme inhibitors or angiotensin-II receptor blockers, beta-blockers, and diuretics. The primary endpoint of the study was postoperative LV reverse remodeling, which was assessed based on the LV end-systolic volume indexed to body surface area (LVESVI) using echocardiography at 6 months after surgery. The secondary endpoints were postoperative changes in LVEF at 6 months after surgery, cumulative survival, and freedom from composite of mortality or readmission for heart failure. Readmission for heart failure was defined as any hospitalization event due to heart failure after surgery. Follow-up was completed in all patients (100%) through a review of their clinical records for a median duration of 3.9 [interquartile range (IQR), 1.8–5.8] years.

### Statistical analysis

The quantitative data were tested for normality with the Shapiro–Wilk test and presented as mean ± standard deviation or median with IQR as appropriate. Normally distributed variables were compared with the Student’s t-test, whereas the Wilcoxon rank-sum test was used for non-normal variables. Categorical variables are shown as frequencies with proportions and were compared using chi-squared analysis or Fisher’s exact test, as appropriate. In view of the selection bias and potential confounding factors, the baseline covariates were adjusted with the propensity score matching. A logistic regression analysis was used to calculate the propensity score for the selection of patients for the single and bilateral ITA groups, using the pre-specified variables, including age, sex, New York Heart Association (NYHA) functional class, chronic kidney disease stage, diabetes mellitus, atrial fibrillation, triple-vessel disease, history of percutaneous coronary intervention (PCI), LVESVI, LVEF, grade of mitral regurgitation, off-pump procedure, and number of distal anastomoses. One patient in the bilateral ITA group was matched to 1 patient in the single ITA group by using nearest-neighbor matching without replacement. The allowable calipers used for the matching included 0.2 SD of the logit-transformed propensity score. A matched balance between the groups was assessed with the standardized mean differences in the variables included in the propensity score estimation. Postoperative changes in LVESVI and LVEF between the study groups were assessed with paired t-test. The associations of preoperative variables with postoperative LV reverse remodeling were examined with logistic regression analysis. The results are summarized as odds ratios (ORs), 95% confidence intervals (CIs), and P-values. Calculation of cumulative survival and the composite of freedom from death and readmission for heart failure were performed using the Kaplan–Meier method, and log-rank testing was performed to compare the groups. The associations of preoperative variables with cumulative survival were examined with Cox proportional hazards analysis. Results are summarized as hazard ratios (HRs), 95% CIs, and P-values. The multivariable model was analyzed using the covariates that demonstrated a P-value of less than 0.1 in the univariable analysis. The number of variables incorporated into the multivariate analysis was confirmed as being appropriate in relation to the total number of events that occurred. Statistical significance was determined as P <0.05. The JMP (Version 17; SAS institute Inc., Cary, NC, USA) software was used for statistical analysis.

## Results

### Patients

The baseline patient characteristics are summarized in **Table 1**. Both groups of patients had identical surgical risks indicated by the logistic EuroSCORE II (2.2% [IQR, 1.5%–3.7%] vs. 3.4% [2.1%–5.5%], P = 0.12). Patients who underwent bilateral ITA grafting were more likely to be younger and to present with less atrial fibrillation and history of PCI when compared with those who underwent single ITA grafting. The bilateral ITA group underwent off-pump surgery more frequently and received a higher number of distal anastomoses. After adjustments for the baseline covariates with propensity score matching, 34 patients were selected from each group. The preoperative characteristics of the 2 groups after matching are detailed in **Table 1**.

**Table 1:** Patient characteristics and surgical data

Variables	All patients			Propensity score-matched patients			
	Single ITA (n = 61)	Bilateral ITA (n = 65)	P value	Single ITA (n = 34)	Bilateral ITA (n = 34)	P value	Standardized mean differences
Clinical data							
Age	70 ± 9	64 ± 10	0.002	68 ± 9	68 ± 9	0.74	0.08
Male, n (%)	56 (92)	61 (94)	0.66	32 (94)	31 (91)	0.64	0.11
Body surface area, median	1.7 ± 0.2	1.7 ± 0.2	0.23	1.7 ± 0.2	1.6 ± 0.2	0.58	0.13
Medical history and presentation at baseline							
Logistic EuroSCORE, median [IQR]	3.4 [2.1–5.5]	2.2 [1.5–3.7]	0.12	2.8 [1.8–4.8]	3.0 [2.0–5.4]	0.70	0.09
NYHA functional class III/IV, n (%)	27 (44.2)	26 (40.0)	0.34	14 (41.1)	13 (38.2)	0.80	0.06
Diabetes, n (%)	42 (68.9)	37 (56.9)	0.17	23 (67.6)	22 (64.7)	0.80	0.06
Chronic kidney disease stage 4 or 5, n (%)	13 (21.3)	8 (12.3)	0.17	5 (14.7)	4 (11.8)	0.72	0.09
Dialysis, n (%)	6 (9.8)	3 (4.6)	0.25	2 (5.9)	2 (5.9)	1.0	<0.001
Atrial fibrillation, n (%)	10 (16.4)	1 (1.5)	0.002	1 (2.9)	1 (2.9)	1.0	<0.001
Peripheral artery disease, n (%)	10 (16.4)	10 (15.4)	0.89	5 (14.7)	7 (20.6)	0.52	0.15
Previous PCI, n (%)	22 (36.1)	11 (16.9)	0.014	5 (14.7)	6 (17.6)	0.74	0.08
Medication at baseline, n (%)							
Beta-blocker	29 (47.5)	34 (52.3)	0.52	18 (52.9)	18 (52.9)	1.0	<0.001
ACE inhibitor or angiotensin receptor blocker	33 (54.1)	37 (56.9)	0.75	22 (64.7)	19 (55.9)	0.46	0.38
Nitroglycerin	18 (29.5)	19 (29.2)	0.97	11 (32.3)	10 (29.4)	0.72	0.06
Coronary lesion							
Triple-vessel disease	50 (82.0)	56 (86.2)	0.52	30 (88.2)	29 (85.3)	0.72	0.09
Left main	17 (27.9)	14 (21.5)	0.41	9 (26.5)	7 (20.6)	0.58	0.14
Proximal left anterior descending	34 (55.7)	36 (55.4)	0.97	21 (61.8)	17 (50.0)	0.33	0.24
Circumflex system	53 (86.9)	60 (92.3)	0.32	32 (94.1)	31 (91.2)	0.64	0.11
Right coronary	52 (85.2)	61 (93.8)	0.11	31 (91.2)	31 (91.2)	1.0	<0.001
Echocardiography at baseline							
LV end-diastolic volume index (ml/m^2^)	112 ± 30	110 ± 26	0.62	112 ± 31	111 ± 29	0.74	0.08
LV end-systolic volume index (ml/m^2^)	80 ± 28	77 ± 23	0.44	78 ± 29	78 ± 26	0.93	0.02
Ejection fraction (%)	29 ± 8	32 ± 6	0.037	31 ± 7	31 ± 7	1.0	<0.001
Moderate mitral regurgitation	6 (10)	1 (2)	0.034	0 (0)	1 (2.9)	0.24	0.24
Surgical data							
Off-pump CABG, n (%)	12 (19.7)	22 (33.4)	0.072	8 (23.5)	10 (29.4)	0.58	0.13
Number of distal anastomoses	3.1 ± 0.9	3.5 ± 0.9	0.024	3.3 ± 0.8	3.4 ± 1.0	0.79	0.07
Bypass configuration, n (%)							
1st ITA target							
Left anterior descending	61 (100)	65 (100)		34 (100)	34 (100)		
2nd ITA target							
Left anterior descending	—	19 (29.2)		—	11 (32.4)		
Circumflex system	—	48 (73.8)		—	24 (70.6)		
Right coronary	—	30 (46.2)		—	11 (32.4)		
Composite grafting (I-composite)	—	21 (32.3)		—	16 (47.1)		
Additional conduit							
Saphenous vein	61 (100)	30 (46.2)		34 (100)	16 (47.1)		
Radial artery	0 (0)	41 (35.3)		0 (0)	18 (52.9)		
Additional conduit target							
Left anterior descending	19 (31.1)	—		11 (32.4)	—		
Circumflex system	45 (73.8)	—		28 (82.4)	—		
Right coronary	50 (82.0)	—		29 (85.3)	—		
All arterial grafting	0 (0)	34 (52.3)	<0.001	0 (0)	17 (50.0)	<0.001	1.39

Values are presented as mean ± standard deviation, median [interquartile range (IQR)], or number (percentage) as shown.

ACE: angiotensin-converting enzyme; CABG: coronary artery bypass grafting; ITA: internal thoracic artery; LV: left ventricle; NYHA: New York Heart Association; PCI: percutaneous coronary intervention

### Postoperative reduction in LV dimension

**Fig. 1:**
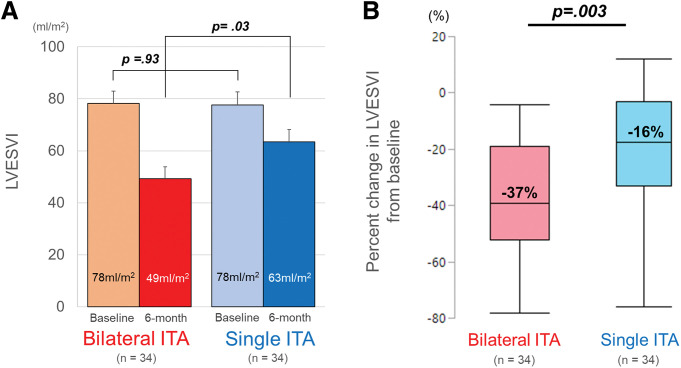
(**A**) Pre- and postoperative changes from the baseline of the LVESVI and (**B**) box-plots showing the percent changes in LVESVI. The box contains the 25th to 75th percentiles of the dataset, with the center line denoting the median value. The whiskers mark the 5th and 95th percentiles. LVESVI: left ventricular end-systolicvolume index

At the preoperative baseline, the mean (±SD) LVESVI was not significantly different between the bilateral and single ITA group (78 ± 26 vs. 78 ± 29 ml/m^2^, P = 0.93). At 6 months, the mean LVESVI was statistically smaller in the bilateral ITA group (49 ± 26 vs. 63 ± 27 ml/m^2^, P = 0.033). The bilateral ITA group reduced LVESVI greater than the single ITA group did (P = 0.005). The median [IQR] absolute change and percent change from baseline were −27 [−42–(−12)] vs. −11 [−24–(−2)] ml/m^2^ and −39% [−52%–(−19%)] vs. −17% [−33%–(−3%)] (**Fig. 1**).

### Predictors of left ventricular reverse remodeling

For the matched cohort, the median [IQR] percent change in LVESVI was −27 [−43–(−11)] ml/m^2^ at 6 months. Therefore, a reduction in LVESVI by ≥27%, derived from the median value of the percentage reduction in LVESVI in the current dataset, was defined as indicative of significant LV reverse remodeling, and 65% (n = 22 out of 34) and 35% (n = 12 out of 34) of patients who underwent CABG with bilateral and single ITA achieved significant LV reverse remodeling, respectively (P = 0.015). Multivariable logistic regression analysis identified that bilateral ITA strategy (OR 3.91; 95% CI 1.38–12.0; P = 0.010) was associated with greater LV remodeling (**Table 2**).

**Table 2:** Logistic regression analysis for postoperative left ventricular reverse remodeling

Variables	Univariable			Multivariable		
	Odds ratio	95% CI	P value	Odds ratio	95% CI	P value
Clinical data						
Age	0.98	0.93–1.03	0.45			
Male sex	1.55	0.24–9.91	0.64			
NYHA functional class III or IV	0.54	0.20–1.44	0.21			
Chronic kidney disease stage 4 or 5	0.77	0.19–3.17	0.72			
Diabetes mellitus	0.88	0.32–2.40	0.80			
Triple-vessel disease	2.21	0.51–9.70	0.28			
Previous PCI	0.31	0.08–1.31	0.09	0.26	0.05–1.09	0.067
Echocardiography						
LV end-systolic volume index	1.00	0.98–1.02	0.91			
LV ejection fraction	0.99	0.92–1.06	0.70			
Surgical data						
Off-pump procedure	1.84	0.61–5.53	0.27			
Number of distal anastomoses	1.71	0.97–3.03	0.06	1.80	0.99–3.53	0.055
Bilateral ITA usage	3.36	1.24–9.09	0.015	3.91	1.38–12.0	0.010

CI: confidence interval; ITA: internal thoracic artery; LV: left ventricle; NYHA: New York Heart Association; PCI: percutaneous coronary intervention

### Postoperative changes in ejection fraction and mitral regurgitation

The LVEF was identical between the groups at baseline (31% ± 7% vs. 31% ± 7%, P = 1.0) but was statistically better in the bilateral ITA group at 6 months postoperatively (44% ± 10% vs. 38% ± 13%, P = 0.032). The improvement in LVEF was significantly better in the bilateral ITA group (P = 0.020). Preoperatively, there was no statistically significant difference in MR severity (1.6 ± 0.7 vs. 1.6 ± 0.7, P = 0.71) and the prevalence of moderate or greater MR (3% (1/34) vs. 0% (0/34), P = 0.24) between the groups. Postoperatively, no difference in MR severity (1.5 ± 0.9 vs. 1.6 ± 0.9, P = 0.68), as well as the prevalence of moderate or greater MR (12% (4/34) vs. 12% (4/34), P = 1.0), was observed between the groups.

### Early and long-term clinical outcomes

**Fig. 2:**
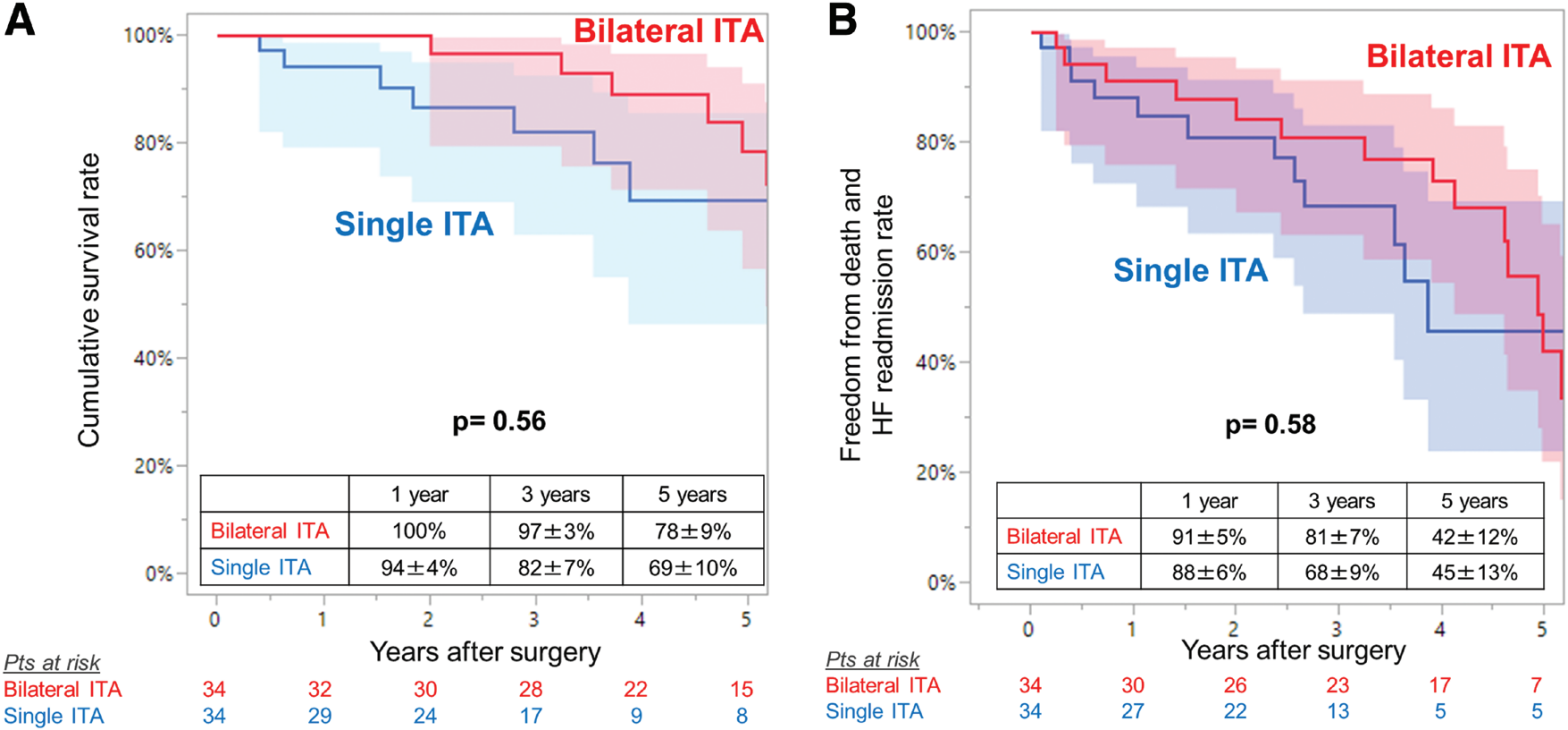
Kaplan–Meier curves for (**A**) overall survival and (**B**) freedom from death and readmission for heart failure in the propensity score-matched cohort (n = 68), with 95% confidence intervals (shaded areas).

During follow-up, 10 (29%) and 9 (26%) died in the bilateral ITA group and in the single ITA group, respectively, and the cumulative survival rates at 1, 3, and 5 years were 100%, 97% and 78%, and 94%, 82%, and 69%, respectively (log-rank P = 0.56). The rates of freedom from death and readmission for heart failure also did not differ between the groups (log-rank P = 0.58) (**Fig. 2**). Univariable Cox proportional hazards analysis identified older age, NYHA functional class III or IV, and chronic kidney disease stage 4 or 5 to be associated with the overall mortality. In contrast, bilateral ITA strategy (HR 0.76; 95% CI 0.30–1.94; P = 0.56) was not associated with cumulative survival (**Table 3**).

**Table 3:** Cox proportional hazards analysis for cumulative survival

Variables	Univariable			Multivariable		
	Hazards ratio	95% CI	P value	Hazards ratio	95% CI	P value
Clinical data						
Age	1.06	1.00–1.12	0.004	1.06	0.99–1.12	0.058
Male sex	2.09	0.28–15.9	0.48			
NYHA functional class III or IV	3.16	1.19–8.35	0.021	1.99	0.65–6.04	0.23
Chronic kidney disease stage 4 or 5	2.89	1.07–7.79	0.036	2.06	0.64–6.57	0.22
Diabetes mellitus	1.42	0.46–4.39	0.54			
Atrial fibrillation	0.99	0.12–7.84	0.99			
Triple-vessel disease	0.76	0.25–2.37	0.64			
Previous PCI	1.54	0.54–4.37	0.42			
Echocardiography						
LV end-systolic volume index	0.99	0.97–1.01	0.48			
LV ejection fraction	1.00	0.93–1.09	0.95			
Moderate mitral regurgitation	2.15	0.28–16.7	0.46			
Surgical data						
Off-pump procedure	1.64	0.23–1.58	0.31			
Number of distal anastomoses	0.87	0.52–1.36	0.56			
Bilateral ITA usage	0.76	0.30–1.94	0.56			

CI: confidence interval; ITA: internal thoracic artery; LV: left ventricle; NYHA: New York Heart Association; PCI: percutaneous coronary intervention

### Relationship between left ventricular reverse remodeling and survival in the bilateral ITA group

**Fig. 3:**
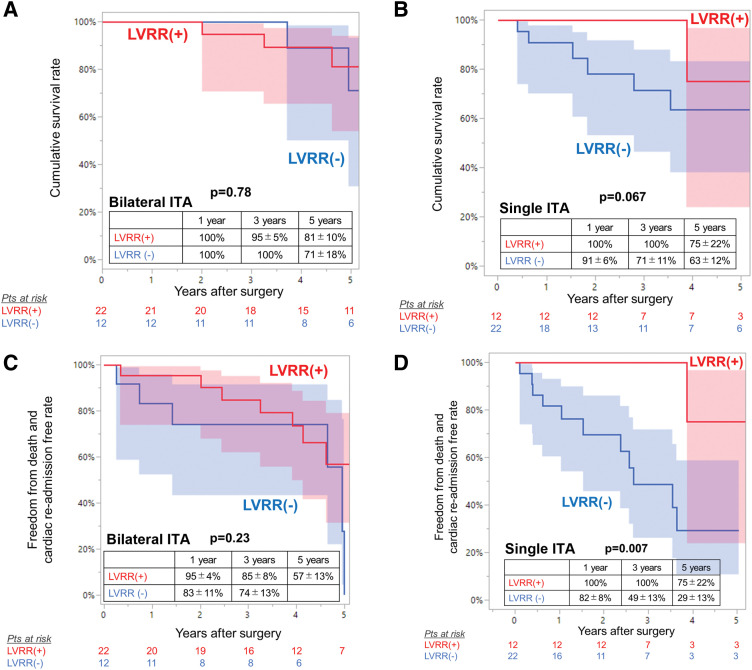
Kaplan–Meier curves for overall survival by postoperative left ventricular reverse remodeling (**A**) in the CABG with bilateral ITA group and (**B**) in the CABG with single ITA group, and for freedom from death and readmission for heart failure by postoperative left ventricular reverse remodeling (**C**) in the CABG with bilateral ITA group and (**D**) in the CABG with single ITA group with 95% confidence interval (shaded areas). CABG: coronary artery bypass grafting; ITA: internal thoracic artery

In the bilateral ITA group, patients who failed to achieve significant LV reverse remodeling showed identical rates of cumulative survival and freedom from composite events compared with those who achieved significant LV reverse remodeling (**Fig. 3**). In the bilateral ITA group, patients who failed to achieve significant LV reverse remodeling showed identical baseline characteristics compared with those who achieved LV reverse remodeling, except for a lower number of distal anastomoses (**Table 4**).

**Table 4:** Patient characteristics and surgical data by postoperative left ventricular reverse remodeling in bilateral and single ITA group

Variables	Single ITA (n = 34)			Bilateral ITA (n = 34)		
	LVRR (n = 12)	No LVRR (n = 24)	P value	LVRR (n = 22)	No LVRR (n =12)	P value
Clinical data						
Age	69 ± 11	68 ± 8	0.69	66 ± 8	70 ± 9	0.17
Male	11 (92%)	21 (95%)	0.66	21 (95%)	10 (83%)	0.25
Body surface area, median [IQR]	1.7 [1.6, 1.9]	1.6 [1.5, 1.7]	0.64	1.6 [1.5, 1.8]	1.6 [1.5, 1.7]	0.44
Medical history and presentation at baseline						
Logistic EuroSCORE, median [IQR]	2.3 [1.1, 3.9]	3.1 [1.8, 6.1]	0.13	3.1 [1.9, 5.4]	2.9 [2.2, 6.1]	0.60
NYHA functional class III/IV, n (%)	3 (25%)	11 (50%)	0.15	8 (36%)	5 (42%)	0.76
Hypertension, n (%)	6 (50%)	17 (77%)	0.11	13 (59%)	7 (58%)	0.97
Dyslipidemia, n (%)	6 (50%)	10 (45%)	0.80	15 (68%)	9 (75%)	0.67
Diabetes, n (%)	7 (58%)	12 (73%)	0.40	15 (68%)	7 (58%)	0.57
Diabetes dependent on insulin, n (%)	1 (8%)	3 (14%)	0.64	5 (23%)	3 (25%)	0.88
Chronic kidney disease stage 4 or 5, n (%)	1 (8%)	4 (18%)	0.42	3 (14%)	1 (8%)	0.64
Dialysis, n (%)	0 (0%)	2 (9%)	0.18	1 (5%)	1 (8%)	0.66
Atrial fibrillation, n (%)	0 (0%)	1 (5%)	0.35	0 (0%)	1 (8%)	0.14
Cerebrovascular disease, n (%)	2 (17%)	4 (18%)	0.91	2 (9%)	1 (8%)	0.94
Peripheral artery disease, n (%)	0 (0%)	5 (21%)	0.028	4 (18%)	3 (25%)	0.64
Previous PCI, n (%)	1 (9%)	4 (18%)	0.42	2 (9%)	4 (33%)	0.08
More than 2 times, n (%)	1 (8%)	1 (5%)	0.66	1 (5%)	2 (17%)	0.25
Medication at baseline, n (%)						
Beta-blocker	7 (58%)	11 (50%)	0.64	11 (50%)	7 (58%)	0.64
ACE inhibitor or angiotensin receptor blocker	9 (75%)	13 (59%)	0.35	13 (59%)	6 (50%)	0.61
Nitroglycerin	4 (3%)	7 (32%)	0.93	4 (18%)	6 (50%)	0.18
Coronary lesion						
Triple-vessel disease	11 (92%)	19 (83%)	0.64	20 (91%)	9 (75%)	0.22
Left main	4 (33%)	5 (23%)	0.51	4 (19%)	3 (25%)	0.64
Proximal left anterior descending	8 (67%)	13 (59%)	0.66	9 (41%)	8 (67%)	0.15
Circumflex system	12 (100%)	20 (91%)	0.18	20 (91%)	11 (92%)	0.94
Right coronary	11 (92%)	20 (91%)	0.94	22 (100%)	9 (75%)	0.009
Echocardiography at baseline						
LV end-diastolic volume index (ml/m^2^)	116 ± 28	111 ± 32	0.68	109 ± 26	114 ± 34	0.65
LV end-systolic volume index (ml/m^2^)	77 ± 24	78 ± 33	0.91	79 ± 26	77 ± 29	0.80
Ejection fraction (%)	33 ± 6	30 ± 6	0.21	30 ± 8	34 ± 4	0.08
Moderate mitral regurgitation	0 (0%)	0 (0%)		1 (5%)	0 (0%)	0.35
Surgical data						
Off-pump CABG, n (%)	1 (8%)	7 (32%)	0.10	6 (27%)	4 (33%)	0.71
Number of distal anastomoses	3.3 ± 0.7	3.3 ± 0.9	0.84	3.6 ± 1.0	2.8 ± 0.7	0.025
Bypass configuration, n (%)						
1st ITA target						
Left anterior descending	12 (100%)	24 (100%)		24 (100%)	12 (100%)	
2nd ITA target						
Left anterior descending				7 (32%)	4 (33%)	0.93
Circumflex system				18 (82%)	6 (50%)	0.055
Right coronary				6 (27%)	5 (42%)	0.40
Additional conduit						
Saphenous vein	12 (100%)	24 (100%)		11 (50%)	5 (42%)	0.64
Radial artery	0 (0%)	0 (0%)		13 (59%)	5 (42%)	0.33
Additional conduit target						
Left anterior descending	5 (42%)	6 (27%)	0.40			
Circumflex system	10 (83%)	18 (82%)	0.91			
Right coronary	11 (92%)	18 (82%)	0.42			
All arterial grafting				11 (50%)	7 (58%)	0.47

Values are presented as mean ± standard deviation, median [interquartile range (IQR)], or number (percentage) as shown.

ACE: angiotensin-converting enzyme; CABG: coronary artery bypass grafting; ITA: internal thoracic artery; LV: left ventricle; NYHA: New York Heart Association; PCI: percutaneous coronary intervention

## Discussion

**Fig. 4:**
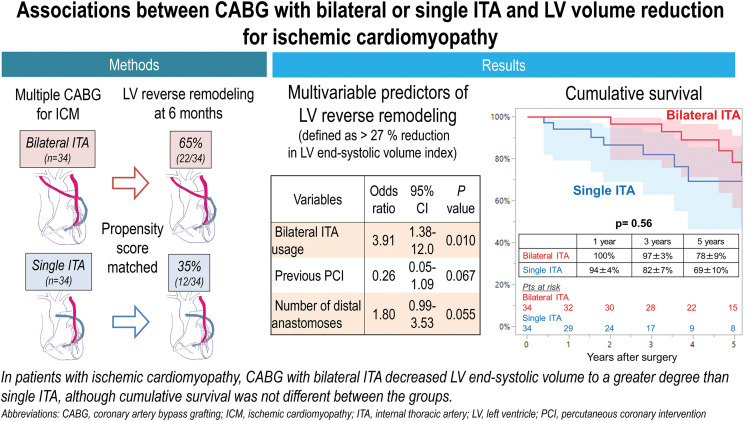
Central illustration depicting the study’s methods, results, and implications.

The major findings of this study can be summarized as follows. In a specific cohort of patients with advanced ICM undergoing multiple CABG, (i) patients who were indicated for the bilateral ITA grafting strategy showed an identical degree of LV remodeling at preoperative baseline. (ii) However, they achieved a greater reduction in LVESVI, yielding significantly smaller LVESVI 6 months postoperatively, compared with those undergoing CABG with the single ITA strategy. (iii) Multivariable analysis showed that the bilateral ITA strategy was associated with a greater reduction in postoperative LVESVI (**Fig. 4**).

Few studies have evaluated the impact of the bilateral and single ITA strategies on LV dimensions and function in ICM. Among the conduits of CABG, the ITA has been shown to produce higher levels of nitric oxide,^8–12)^ rendering it less susceptible to atherosclerotic change.^7)^ As well as protecting the conduit from atherosclerotic change, nitric oxide has also shown beneficial effects on ventricular reverse remodeling and cardiac functional recovery in ICM.^13–16)^ As the increased production of nitric oxide from the ITA is confirmed not only in the conduit itself but also in the distal recipient coronary artery,^18)^ it is speculated that the utilization of bilateral ITA supplies increased nitric oxide to the global cardiac vasculature compared with single ITA. In our study, patients undergoing CABG with bilateral ITA achieved significantly greater reduction in LV volume, as measured by LVESVI, than did those undergoing CABG with single ITA. So far, limited data are available regarding postoperative LV reverse remodeling after CABG, especially for patients with ICM. In the Cardiothoracic Surgical Trials Network group study for moderate ischemic MR, Smith et al. randomly assigned 301 patients (mean LVEF 40%, LVESVI 75 ml/m^2^) to CABG with mitral repair or CABG alone.^19)^ One year postoperatively, the CABG-alone group reduced LVESVI by 17% (absolute reduction; 9.4 ml) from baseline. In the STICH (Surgical Treatment for Ischemic Heart Failure) trial reported by Jones et al., 1000 patients (mean LVEF 28%, LVESVI 86 ml/m^2^) were randomly assigned to CABG with surgical ventricular restoration or CABG alone. The CABG-alone group reduced LVESVI by 6% (absolute reduction: 5 ml) at 4 months postoperatively.^20)^ Our results from patients with single ITA strategy (percent change in LVESVI −17%) appear consistent with the results from Smith et al. regarding the degree of reduction in LV dimension. It is worth noting that the patients enrolled in our study presented more advanced stages of LV remodeling, as indicated by the larger LV volume despite less severity in MR, than those enrolled in the study by Smith (e.g., LVESVI; 78 vs. 56 ml/m^2^). In terms of baseline LV remodeling, our results appear to be consistent with results from Jones (baseline LVESVI 78 vs. 82 ml/m^2^). However, postoperative LV reverse remodeling seems greater in our study (percent change in LVESVI 27% vs. 6%). The rate of triple-vessel disease seems less for the Jones’s cohort (64% vs. 87%), which can be accounted for by a smaller number of target vessels in CABG. Notably, the bilateral ITA group in our study showed greater LV reverse remodeling compared to previous studies (percent change in LVESVI 39%). Direct comparison of the findings among the studies may be difficult due to baseline differences in patient characteristics, including LV functions and MR grade, and surgical strategies, such as conduit selection. However, multivariable analysis in our study confirmed that bilateral ITA strategy was associated with greater reduction in LVESVI, suggesting potential benefit of bilateral ITA strategy for postoperative LV reverse remodeling in patients with advanced ICM. Certainly, this study did not evaluate biological parameters such as nitric oxide production associated with single or bilateral ITA use. Therefore, the impact of ITA strategies on postoperative left ventricular function remains speculative and requires further investigation.

In terms of survival benefits of the bilateral ITA strategy, controversy still remains, though there is accumulating evidence in patients with preserved LV systolic function who undergo isolated CABG. In cases with preserved LVEF, surgical revascularization can be performed generally with a low surgical risk, irrespective of the graft strategy, and noticeable differences in the outcomes were not observed within the early follow-up period. However, in the long term, the superiority of the graft patency in CABG with bilateral ITA strategy becomes prominent, which possibly translates into lower risk of coronary events and better mortality rates in favor of the bilateral ITA group.^21–25)^ In patients with impaired LV function, to the contrary, little is known about survival benefits of the bilateral ITA strategy. To primarily focus on the relationships between postoperative LV reverse remodeling and long-term clinical outcomes, our study included only patients who completed postoperative echocardiography at 6 months and thus eliminated the effect of early perioperative mortality on long-term results. Among our series of patients, bilateral ITA strategy was associated with improved postoperative LV function; however, no significant association was observed with all-cause mortality or hospitalization for heart failure. Further research is required to clarify the extent to which improvements in LV function translate into prognostic benefit in patients with advanced ICM. Our results might be supported by findings from Galbut et al. regarding an analysis of propensity score-matched 174 patients who underwent CABG with bilateral or single ITA (age 64 years, LVEF <30%).^26)^ No intergroup difference was observed in mortality; 4-year survival rates were 70% for both the bilateral and single ITA groups, which were comparable to the survival rates of the bilateral (78%) and single ITA group (69%) at 5 years in our study. Our results were also consistent with reports from Mohammadi et al.^27)^ They analyzed 222 propensity-matched patients with low ejection fraction (mean EF 33%) undergoing isolated primary CABG with bilateral or single ITA, resulting in no intergroup difference in late survival. Although, 5-year survival rates for both groups (94% and 83% for the bilateral and single ITA groups) seem better than our results, presumably because they included younger patients (mean age, 56 years vs. 68 years) with lower prevalence of heart failure (NYHA functional class III/IV 23% vs. 40%) than our cohorts. Nevertheless, the impact of bilateral ITA strategy on long-term survival in patients with advanced ICM remains to be determined.

### Limitations

There are some limitations to our study. First, this study was retrospective in nature and included a small number of subjects from a single center; thus, our results should be interpreted cautiously until verified in an independent, prospective study. We assessed LV volume reduction via LVESVI obtained by echocardiography, which may render imprecise volumes. More precise analyses can be accomplished with detailed imaging modalities such as computed tomography or magnetic resonance imaging. Although myocardial viability is a key factor in LV reverse remodeling, it was not available in our database. In this study, revascularization was performed in all 3 coronary artery territories in over 80% of cases in both groups. Therefore, it is inferred that there is no significant difference in viability between the 2 groups at the level of the major coronary artery territories. Although guideline-directed medical therapy is known to play an important role in left ventricular reverse remodeling, the impact of postoperative medication could not be evaluated in this study due to the absence of postoperative medication data in our surgical database. There may be factors not currently captured in the database that could influence CABG graft selection and postoperative left ventricular reverse remodeling. For example, whether a suitable coronary artery target exists for the second ITA is an important consideration. Unfortunately, this database does not provide detailed information on the coronary artery targets for individual cases. We employ a strategy utilizing sequential bypass with right ITA–radial artery I-composite grafts (used in 32% of the bilateral ITA group) to achieve as much bypass grafting as possible with the in situ right ITA as the inflow source for non-left anterior descending targets. This approach allows us to maximize arterial grafting options, supporting the use of right ITA in a broader range of cases. Also, era-related bias cannot be fully excluded because this study included patients who underwent surgery over an extended time period. Changes in perioperative management, surgical techniques, and medical therapy throughout the study period may have influenced clinical outcomes. Finally, further investigation of a larger patient population with a longer follow-up is required to definitively confirm our results.

## Conclusion

In patients with ICM undergoing CABG, bilateral in situ ITA strategy was associated with greater reductions in postoperative LV volume compared to CABG using a single in situ ITA and venous conduits. Further study is warranted to elucidate the impact of bilateral ITA strategy on postoperative outcomes.

## IRB Information

This study was conducted with patient data retrieved from the Osaka Cardiovascular Research Group (OSCAR) database, which was approved by the institutional ethics committee of Osaka University Hospital, and written informed consent from each patient was waived for this retrospective study (Reference no. 08218-6).

## Declarations

### Data availability

The deidentified participant data will be shared on a request basis. Please directly contact the corresponding author to request data sharing.
